# Efficacy of Bovine Nail Membranes as In Vitro Model for Onychomycosis Infected by *Trichophyton* Species

**DOI:** 10.3390/jof8111133

**Published:** 2022-10-27

**Authors:** Marta Elisabetta Eleonora Temporiti, Marta Guerini, Rebecca Michela Baiguera, Simone Buratti, Anthea Desiderio, Lorenzo Goppa, Paola Perugini, Elena Savino

**Affiliations:** 1 Department of Earth and Environmental Sciences (DSTA), University of Pavia, Via Sant’Epifanio 14, 27100 Pavia, Italy; 2 Department of Drug Sciences, University of Pavia, Via Taramelli 12–14, 27100 Pavia, Italy

**Keywords:** onychomycosis, *Trichophyton*, dermatophytes, bovine nail membranes

## Abstract

Onychomycosis is a fungal infection caused by different etiologic agents, including dermatophytes that specifically colonize keratin-rich substrates. The aim of this work was to investigate mechanical modifications of bovine membranes (used as an in vitro nail model) placed in contact with *Trichophyton* species. *Trichophyton* strains were isolated from toenails specimens. The procedure was set up by spreading *T. rubrum,* *T. interdigitale,* and *T. mentagrophytes* strains on Petri dishes with minimal and rich media; after that, bovine membranes were placed in the center. After 27 days, *T. interdigitale* and *T. mentagrophytes* significantly reduced the thickness of the colonized membranes, whereas two *T. rubrum* strains showed the highest degradation limited to the small colonized area. These results were confirmed by SEM images of the colonization profile on membranes. Mechanical analyses performed on membranes were used as an innovative method to evaluate the thickness and structural integrity of membranes variation following fungal colonization. In conclusion, mechanical analyses of substrate may be used as a procedure for the development of a new onychomycosis diagnosis test in order to develop personalized and strain-specific treatment.

## 1. Introduction

Onychomycosis is a fungal infection affecting nails caused by yeasts (mainly belonging to the genus *Candida*), dermatophytes (mainly *Trichophyton* species), and molds (such as *Aspergillus, Fusarium,* and *Scopulariopsis* species) or from the combination of more than one pathogen [1,2,3]. Development of onychomycosis is influenced by endogenous and exogenous factors: comorbidity with other pathologies such as diabetes mellitus, obesity, and aging and by the geographic area [4,5,6,7,8]. For all these reasons, onychomycosis is considered a multifactorial disease [9]. Infection is transmitted through the nail’s direct contact with dermatophytes, molds, or yeasts. Onychomycosis is difficult to treat, and relapses and reinfections are common because the nail unit does not have effective cell-mediated immunity [4,8].

The first clinical classification for the diagnosis of onychomycosis was proposed in 1972 [7]. Current classification has five different clinical subtypes: White Superficial Onychomycosis, Distal Lateral Subungual Onychomycosis, Proximal Subungual Onychomycosis, Endonyx Onychomycosis, and Total Dystrophic Onychomycosis [4,8,10]. A useful diagnosis can be made when both positive laboratory and clinical criteria are present [11]. If untreated, onychomycosis may lead to the destruction of the nail plate, ingrown nails, poor nail formation, secondary infections, and also psychological distress [1].

Distribution of etiological agents of onychomycosis changes depending on geographical areas. In southern European countries, dermatophytes resulted in being responsible for 40–68% of onychomycosis, and 21–55% were caused by yeasts [12]. In North America, dermatophytes reached 80–90%, yeasts 5–17%, and 2% are caused by non-dermatophytic molds [1]. In Asian and Middle Eastern countries, dermatophytes caused 40–48% of onychomycosis, yeasts 43–46%, and non-dermatophytic molds 8–11% [9,12].

Dermatophytes are a group of filamentous fungi able to specifically degradate keratin, so they can colonize the keratinized tissue of mammals, becoming parasitic to animals and humans. Species belonging to this group of fungi are adapted to particular ecological niches and hosts, which leads to the classification of anthropophilic, zoophilic, and geophilic fungi. Generally, anthropophilic species such as *Trichophyton rubrum* (Castell.) Sabour. Blanch and *Epidermophyton floccosum* (Harz) Langeron and Miloch invade nails or human skin causing infection with mild clinical symptoms [13,14,15].

Instead, zoophilic and geophilic species are more frequently isolated from patients suffering from mild to highly inflammatory dermatophytosis [2,13,15]. The main characteristic of this fungal group is the production of enzymes with specific keratinolytic activity. Degradation of keratin at the nail plate facilitates the invasion of dermatophytes into the nail [4,16]. The process of keratinolysis involves the cooperative action of sulfitolytic and proteolytic systems. Enzymes mainly produced are keratinases, including serine- or metallo-proteinases [14,17,18]. A significant feature of keratin structure consists in being largely composed of sulfide bridges due to the presence of cysteine and methionine [19,20]. The first phase of keratinolysis is sulfitolysis. In this phase, the extensive disulfide bridges present in keratin are hydrolyzed, simplifying the extracellular biodegradation of keratin by the dermatophytes’ extensive array of endo- and exo-proteases. Finally, the short peptide and aminoacid breakdown products are taken up by the dermatophyte. The importance of dermatophyte proteases in infection is widely recognized, and these enzymes have also been identified as important virulence determinants and allergens [17,18,19,20].

Onychomycosis is caused mainly by antropophilic and zoophilic *Trichophyton* species. *T. rubrum* and *Trichophyton mentagrophytes* (C.P. Robin) R. Blanch account for more than 90% of onychomycosis [11,12]. Specifically, 71% from *T. rubrum* and 21% from *T. mentagrophytes*. This species is closely related to *Trichophyton interdigitale* (Jacz)*,* and they are also indicated as *T. mentagrophytes/T. interdigitale* complex, but their taxonomy is still debated [21,22,23]. *T. interdigitale* may cause superficial infections in humans and other mammals, specifically on nails [23,24,25].

Over the years, due to the difficulty of finding effective solutions to treat onychomycosis, in vitro models that can simulate the human nail have been developed [26].

In literature, the most used nail models are cadaver nails, human nail clippings, and bovine hoof membranes [26,27,28]. Cadaver nails are the least used model because nail recovery takes longer and, more importantly, requires bureaucratic approval that can significantly delay the research process. Human nails and animal hooves are composed of the same type of keratin (alpha keratin). The relationship between aminoacidic groups and their internal structures are the main differences. Furthermore, they both have a high content of fundamental sulfide groups to ensure extreme keratin hardness [29,30].

The purpose of the present work was to investigate mechanical modifications of bovine membranes (used as an in vitro nail model) placed in contact with the mycelium of *Trichophyton* species. The *Trichophyton* strains tested were previously isolated from toenail specimens. Bovine membranes were used as an in vitro model both to mimic the natural condition of nails and to assess the efficacy of this nail model as an in vitro model for onychomycosis. The production and characterization of bovine membranes were previously studied and standardized [29]. For this purpose, it is possible to produce bovine membranes with thicknesses and mechanical characteristics suitable for specific studies. In previous works, it was observed that membranes with thicknesses around 200 μm are used for permeability studies, whereas those with thicknesses equal to or greater than 400 μm (more like human nails) are used for in vitro product efficiency and safety analysis prior to in vivo application.

Our work is intended to be a starting point for the definition of a new test for in vivo diagnosis and for personalized therapy too. This could be realized thanks to the possibility of combining microbiological assessment with an innovative apparatus measuring mechanical characteristics of tissue that can be applied both on membranes coming from cattle and directly in vivo on patients.

## 2. Materials and Methods

### 2.1. Collection of Nail Samples Affected by Onychomycosis

Patients with suspected onychomycosis provided nails after cutting and scratching the surface with a sterile scalpel [30]. Samples were then stored in sterile containers and taken to the Mycology laboratory of the DSTA (University of Pavia, Italy). Before sampling, nails were required not to be treated with any antifungal for at least fifteen days or with nail polish for at least one week. When the samples were brought to the laboratory, identification codes were assigned, and they were placed in a cultural medium for etiological agents’ isolation as soon as possible.

### 2.2. Isolation of Etiological Agents

Nail samples were inoculated into Petri dishes containing Sabouraud Dextrose Agar (SDA) with 50 ppm chloramphenicol, Dichloran Rose Bengal Chloramphenicol (DRBC), and Dermatophyte Selective Medium (DSM). All the culture media were autoclaved at 121 °C, 1 atm for 20 min. The material to be analyzed was inoculated with a three-points lightly submerged in the Petri dishes medium, keeping enough distance among them to avoid overlap of each growth. The procedure was performed in triplicate for each culture medium. Petri dishes were then incubated at 25 °C for one month. Grown fungal strains were isolated on SDA tubes and incubated at 25 °C for one week in order to obtain pure isolates.

### 2.3. Fungal Identification

Isolated pure cultures were identified by morpho-dimensional examination under an optical microscope. The dyes Amman’s Lactophenol with acid fuchsin were used to analyze the micro-morphological characteristics of each strain [31,32].

ITS rDNA molecular characterization was performed to achieve a correct identification. Pure fungal strains were lyophilized to favor homogenization, and then fungal genomic DNA was extracted using NucleoSpin Plant II by Macherey-Nagel (Bethlehem, PA, USA). After extraction, Polymerase Chain Reaction (PCR) amplification of the internal transcribed spacer (ITS) region of the ITS1 (5′-TCCGTAGG TGAACCTGCGG-3′) and ITS4 (5′-TCCTCCGCTTATTGATATGC-3′) rDNA gene was performed using Green Taq Mastermix (Promega, Milano, Italy) in a Thermocycler Bio-Rad T100 [33]. Amplification product purification was carried out by ExoSAP-IT (Applied Biosystems, Foster City, CA, USA), according to the suggested protocol. PCR reaction was performed as follows: denaturation by heating for 5 min at 95 °C, then 35 cycles of 30 s at 95 °C, 45 s at 50 °C, 1 min at 72 °C and a final elongation step for 10 min at 72 °C in the thermocycler. Purified DNA was sent to Macrogen (The Netherlands), and the sequence analysis was performed by Sequencher 5.0 Demo. Finally, sequences were matched with the ones available in the molecular identification facility of Mycobank [34].

For this study, only dermatophytes identified at the species level were used to evaluate the degradation of bovine membranes employed as nail models.

### 2.4. Growth Tests

To perform subsequent experiments on bovine membrane colonization, identifying the best cultural media was necessary. Therefore, three types of culture media with different nutrient concentrations were tested. Media were prepared as follows:

- Water Agar (WA) as a minimal medium: 15 g/L Agar;
- Sabouraud Dextrose Agar (SDA) as rich medium: 30 g/L Sabouraud Dextrose Broth + 15 g/L Agar;
- ½ SDA as an intermediate medium: 15 g/L of Sabouraud Dextrose Broth and 15 g/L Agar.

Cultural media were poured into 60 mm Petri dishes.

Fungal suspension of each dermatophytes strain was performed by collecting actively growing mycelium in test tubes containing broken coverslips and 10 mL of distillate water. Tubes were vortexed for 2 min to obtain a fungal suspension of 1 × 10^6^ CFU/mL (colony-forming unit/mL). Then, for each strain, 20 µL of fungal suspension was inoculated in the center of the Petri dishes. All operations were carried out under sterile conditions, and tests were replicated three times for each fungal strain and cultural medium. Petri dishes were incubated at 30 °C for 14 days [35,36]. During the incubation, the growth radius was measured five times up to a total growth of 14 days, which was the maximum colonization of the Petri dishes.

### 2.5. Bovine Membrane Production

Employed membranes were obtained from freshly slaughtered 3-years-old cattle (Azienda Agricola Pluderi Marcellino, San Colombano al Lambro, Italy). Freshly slaughtered bovine hooves were dipped in liquid nitrogen, cored using a Rolson^®^ plug cutter with a 16 mm diameter from Rolson Tools Ltd. (Twyford, UK), and cut into slices of about 800 μm with a Graziano SAG 12 precision lathe (Tortona, Alessandria, Italy). The liquid nitrogen was essential to prevent mechanical deformation during core drilling and subsequent cutting. For the experiments, 28 membranes were chosen, and a suitable sanitization protocol was applied to start with very low microbial content, using a sequence of washing with ethanol 70% *v/v* and a benzalkonium chloride mixture (0.4 g benzalkonium chloride, 70 g isopropyl alcohol, distilled water to 100 g). Membranes were then maintained in a climatic chamber (ClimaCell 111, MMM Medcenter Einrichtungen GmbH, Munchen, Germany) at 25 °C and 40% Relative Humidity (RH).

### 2.6. Fungal Colonization of Bovine Membrane

Briefly, 500 µL of each fungal suspension (1 × 10^6^ CFU/mL) was inoculated and spread in 90 mm Petri dishes containing WA or ½ SDA. Sanitized bovine membranes were placed in the center with sterilized tweezers and then incubated at 30 °C. The first visible contact and subsequent growth on the membranes were observed regularly, until the complete membranes colonization, by a stereomicroscope. It is considered the first visible contact the observation of mycelium grown from Petri dishes towards the membranes. To evaluate the percentage of colonization by fungi, the membranes were photographed at the same height so that the images could be compared. Subsequently, the images were imported onto PowerPoint and compared with a grid that was affixed to them, so it was possible to calculate the percentage coverage. In addition, further validation from the coverage percentage was given using ImageJ software (Software version 1.53t, National Institute of Health, Maryland, USA).

### 2.7. Membrane Characterization

#### 2.7.1. Contact Angle Measurement

The procedure to measure contact angle (CA) was previously reported [29]. Membranes, before and after fungal colonization, were characterized with Contact Angle Meter DMe-211Plus (KYOWA) using 10 µL of MilliQ^®^ water. Membranes with CA < 65° are considered wettable/hydrophilic, with CA > 65° poorly wettable/hydrophobic [32,35]. Moreover, CA percentage variation over time (from the deposition time of the drop to 40 s) was evaluated.

#### 2.7.2. Mechanical Properties Assessment

Mechanical characterization was carried out using Nail StrainStress Meter-NM100, Courage and Khazaka Electronic GmbH [37]. Thanks to this instrument, it was possible to measure the thickness and compactness of the nail before and after the fungal colonization. Firmness refers to how cohesive the corneocytes that make up the nail plate are. This parameter is referred to as the Structural Strength or Firmness Index (FI). To have the membrane in a valid position for the mechanical evaluation, which should mimic as much as possible a real in vivo situation, samples were mounted on custom-made PTFE supports. Then, to minimize the influence of membrane length on the analysis, the position of the membrane on the holder was standardized to 5 mm. Precise parameters were set before the analysis (Table 1).

#### 2.7.3. Scanning Electron Microscopy

Microstructural characterization was performed with a high-resolution Scanning Electron Microscope (TESCAN, Mira 3 XMU) equipped with an In-Beam SE detector and operated at 5 kV. The samples were previously mounted on aluminum pin stubs by graphite tape and coated with graphite using a Cressington 208HR.

### 2.8. Statistical Analysis

One-way analysis of variance (ANOVA) was performed to compare multiple groups; two samples paired *t*-test was used to compare the data before and after fungal colonization, and differences were significant for *p* < 0.05. All growth and membrane colonization tests were performed in triplicate.

## 3. Results

### 3.1. Dermatophytes Isolation and Identification

**Figure 1:**
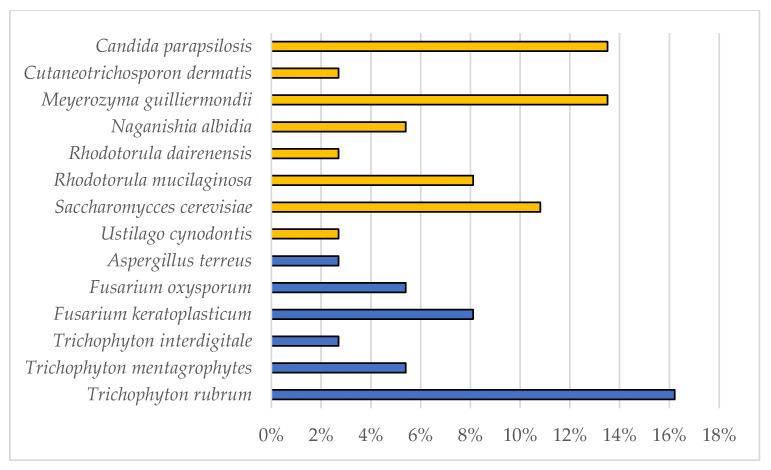
Percentage of etiological agents of onychomycosis isolated at the Mycology Laboratory of DSTA (UNIPV, Italy). Filamentous fungi are indicated with blue bars, whereas yeasts with yellow bars.

Since September 2020, 96 nail samples were examined at the Mycology Laboratory of DSTA (UNIPV, Pavia, Italy) with a positivity rate of 42%. From the 40 positive samples, through macro- and micro-morphological observations and molecular analysis, 24 yeasts, 9 dermatophytes, and 7 other species of filamentous fungi were isolated and identified (Figure 1).

As concerns dermatophytes, all the strains were isolated from toenails and belonged to the genus *Trichophyton*: 6 *T. rubrum*, 2 *T. mentagrophytes,* and 1 *T. interdigitale* (Table 2).

### 3.2. Growth Tests

**Figure 2:**
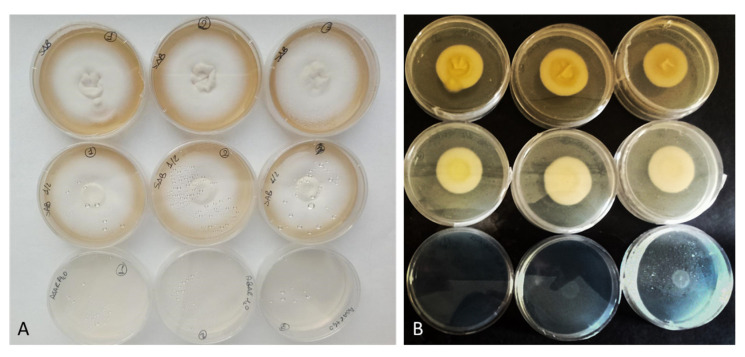
Growth tests of *T. interdigitale* 10379 with SDA (first line), ½ SDA (second line), and WA (third line) after 14 days of incubation at 30 °C (frontal view) (**A**) and after 7 days at 30 °C (back of Petri dishes) (**B**).

Growth of the 9 *Trichophyton* strains was evaluated in 3 different cultural media with different nutrient supplies: WA, SDA, and ½ SDA. WA was chosen to reduce the influence of nutrients in the culture medium and stimulate the colonization of the fungus on bovine membranes in the subsequent tests. The *T. interdigitale* strain 10379 growth tests were reported as an example (Figure 2).

After 14 days, *T. rubrum* strains reached 1.5 cm in WA, except strain 10351, which showed maximum growth of 3.5 cm. The same value was reached by *T. interdigitale* and *T. mentagrophytes* in WA. A different trend was observed when the strains were grown on rich media.

In all the tested strains, mycelium was very underdeveloped; the hyphae were rare and thinned out in WA, whereas the mycelium in the rich medium was fluffy, cottony, and well-developed. The growth trend in SDA is almost linear for all the tested strains (R^2^ > 0.986) except for strain 10318, which showed a decrease in growth rate between day 10 and day 14 (Figure 2).

As there was a low difference between SDA and ½ SDA, the former (culture medium with the highest nutrient concentration) was excluded from the subsequent tests.

Differences in the growth radius of *T. rubrum* strains 1000A and 1000B between WA medium and SDA medium were statistically significant (ANOVA; *p* < 0.05), whereas in strain 10318, the only difference between WA and ½ SDA resulted statistically significant (ANOVA; *p* < 0.05).

**Figure 3:**
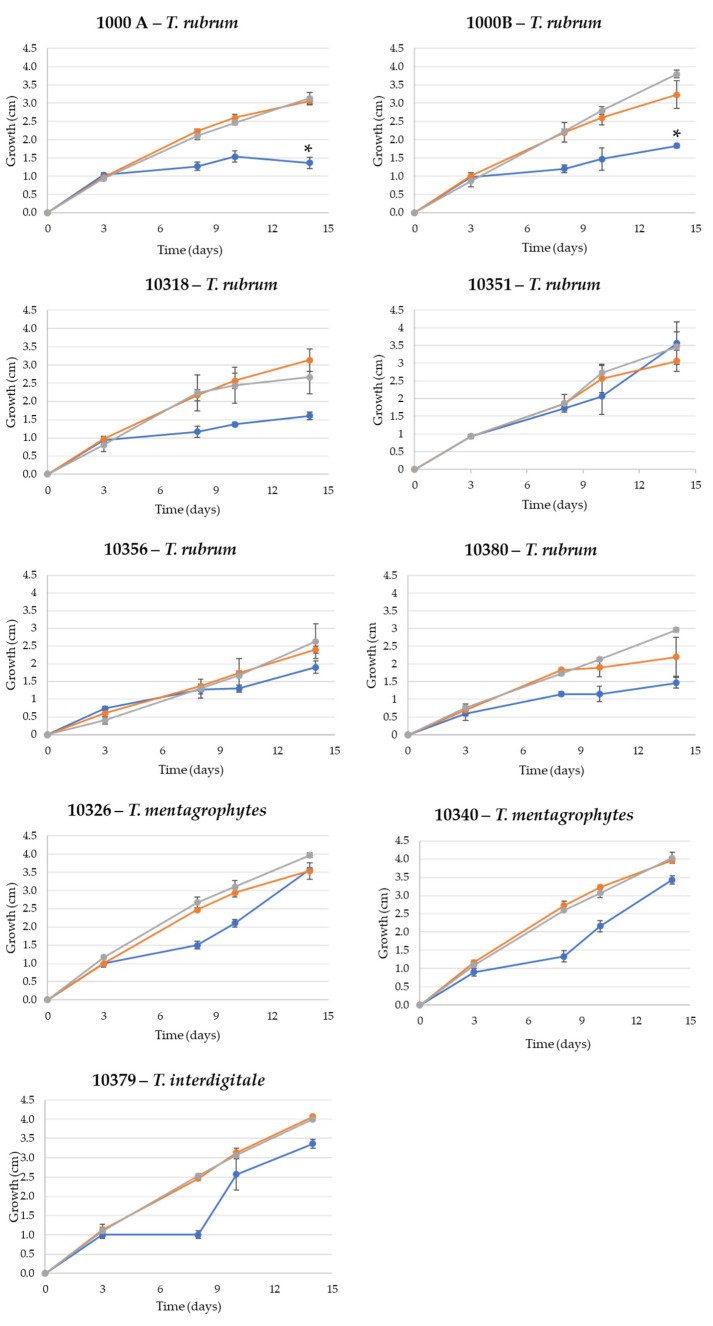
Growth tests of *T. rubrum* (1000A, 1000B, 10318, 10351, 10356, and 10380), *T. mentagrophytes* (10326 and 10340), and *T. interdigitale* (10379) in WA (blue), ½ SDA (orange), and SDA (grey). Error bars indicate the standard deviation of the three replicates used for the tests. ANOVA tests were performed, and statistically significant differences are marked with *.

The *T. rubrum* strains 10356 and 10380 showed a very slow growth with a maximum hyphal elongation minus of 3 cm in both rich and minimal media after an incubation period of 30 days (a longer time was allowed to verify the actual growth) (Figure 3).

### 3.3. Membrane Colonisation

In vitro tests on the bovine membrane were performed to evaluate the different nail colonization abilities of the nine *Trichophyton* strains. Moreover, differences between growth in ½ SDA and WA were observed.

At first, it was observed whether the strains contacted the bovine membranes and then if they colonized them.

**Figure 4:**
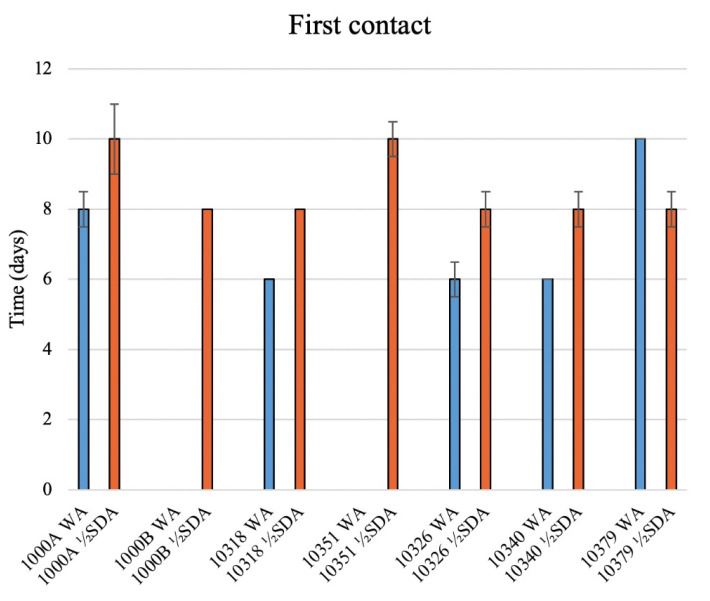
Time (days) elapsed since first visible contact with membrane of 7 dermatophyte strains in WA (light blue) and ½ SDA (orange). Error bars indicate the standard deviation of the three replicates used for the tests.

Only seven out of nine strains showed a first visible contact with bovine membranes: *T. rubrum* 10356 and 10380 did not get any contact (data not shown in Figure 4).

**Figure 5:**
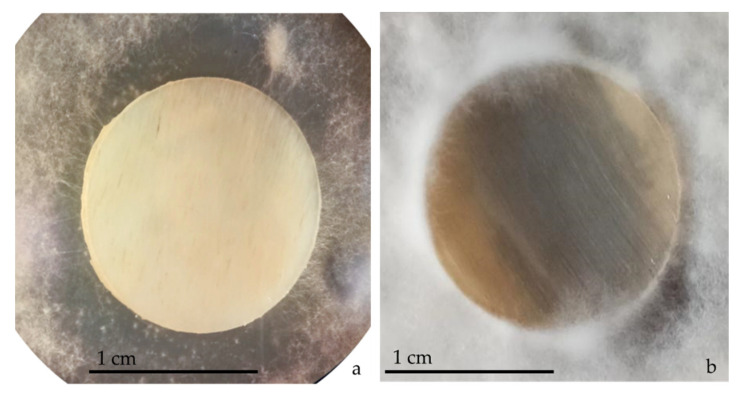
*T. mentagrophytes* 10340 first visible contact with bovine membrane in WA medium at day 6 (**a**), and *T. interdigitale* 10379 grown in ½ SDA initial colonization (**b**).

The first visible contact of dermatophytes with bovine membranes occurred within 10 days, regardless of the culture medium used (Figure 4; SD always <5%). In WA, *T. mentagrophytes* strains (10326 and 10340) established the first visible contact in about 6 days, whereas *T. interdigitale* 10379 needed 10 days (Figure 5). *T. rubrum* strains 10318 and 1000A reached the membrane in 6 and 8 days, respectively, whereas *T. rubrum* strains 1000B and 10351 were not able to make any contact with the membranes in the minimal medium (WA). In contrast, in ½ SDA, all these seven strains established the first visible contact in 8 days, except for 1000A and 10351, which needed 10 days.

Therefore, considering obtained results, *T. rubrum* 10356, 10380, 1000B, and 10351 were excluded from subsequent analyses regarding membrane characterization after fungal growth.

**Figure 6:**
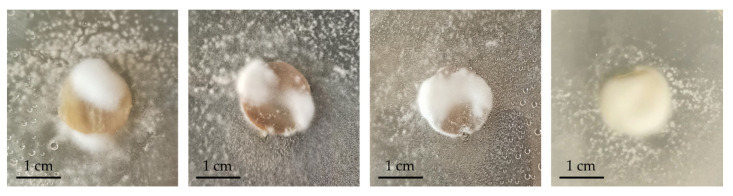
*T. interdigitale* 10326 growth on bovine membrane in WA at 10, 12, 15, and 27 days (from left to right).

**Figure 7:**
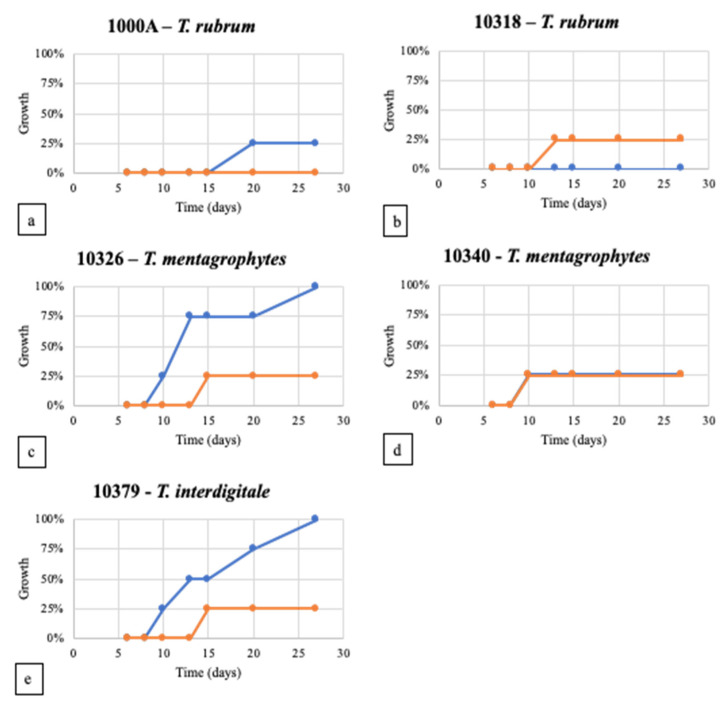
Membrane coverage percentage by *T. rubrum* 1000A (**a**) and 10318 (**b**), *T. mentagrophytes* 10326 (**c**), *T. mentagrophytes* 10340 (**d**), *T. interdigitale* 10379 (**e**) in WA (blue line) and ½ SDA (orange line).

Initial mycelium development on the membrane surface, referred to as colonization, was then examined (Figure 6 and Figure 7). Results showed a low percentage of colonization (25%) for *T. rubrum* strains: 1000A was able to colonize the membranes after 20 days only in WA, whereas 10318 after 13 days in ½ SDA. The two other *T. rubrum* strains were not able to grow on bovine membranes (data not shown in Figure 7). On the other hand, all the strains tested belonging to *T. interdigitale* and *T. mentagrophytes* colonized the membranes after 10 days in WA and between 10 and 15 days in ½ SDA. The strains 10379 (*T. interdigitale*) and 10326 (*T. mentagrophytes*) were able to cover 100% of the membrane surface in WA medium after 27 days, whereas in ½ SDA, they reached a plateau in 15 days by covering only 25% of the membrane surface. *T. mentagrophytes* 10340 showed the same behavior in WA and ½ SDA, covering 25% in 10 days, but then colonization never continued.

### 3.4. Membrane Characterization

#### 3.4.1. Contact Angle Measurement

**Figure 8:**
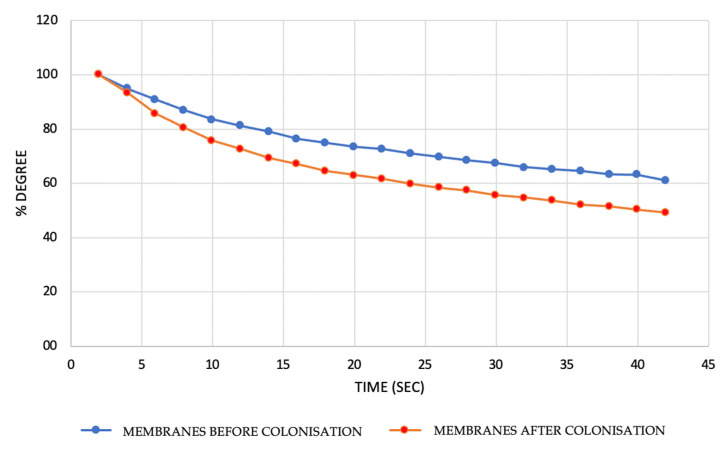
Percentage decrease in the CA value of membranes before and after fungal colonization.

To evaluate the wettability properties of the bovine membranes with water, a total of 30 samples, before and after fungal colonization, were analyzed. The first measurement (t0) of the contact angles (CA) was performed after 2 s from the fall of the drop on the membrane. Before and after fungal colonization, the CA resulted in similar (71.08 ± 12.07 and 81.06 ± 16.95, respectively, *p* > 0.05). Therefore, all the tested bovine membranes (with or without fungi) were poorly wettable. The wettability trend over time was also measured regularly for 40 s. Therefore, the percentage decrease in CA value was used to make the trend independent of absolute values. The graph in Figure 8 shows that the membranes had the same trend.

#### 3.4.2. Mechanical Properties Assessment

**Figure 9:**
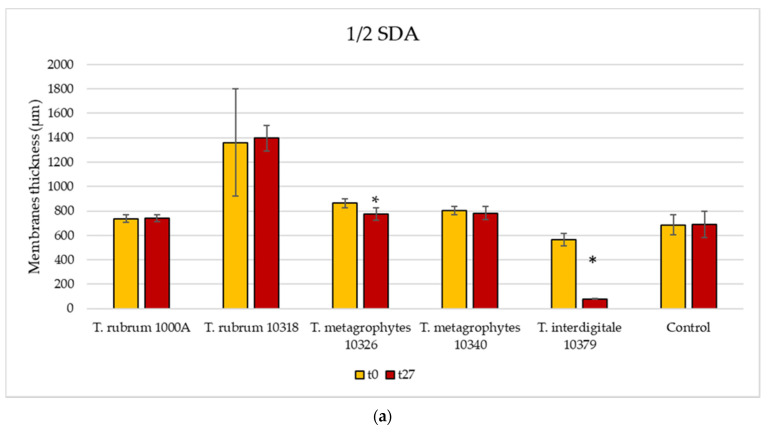

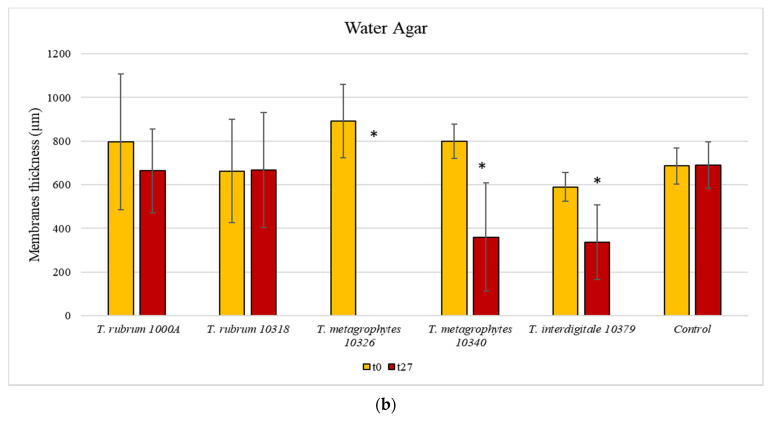
Membrane thickness at time zero (t0) and after 27 days of fungal colonization (t27) in ½ SDA (**a**) and in WA (**b**). Error bars indicate the standard deviation of the three replicates used for the tests. Two-sample paired *t*-test were performed, and statistically significant differences are marked with *.

The thickness of the ungual bovine membrane colonized by the fungi was measured to evaluate modification due to fungal activity. Results showed that, generally, there was a greater decrease in the thickness of membranes placed in WA than in ½ SDA (Figure 9a,b). The strains *T. interdigitale* 10379 and *T. mentagrophytes* 10326 colonized the membranes in both rich and minimal media, resulting in a statistically significant decrease in thickness (two-sample paired *t*-test; *p* < 0.05). It must be noted that the latter was the only one that in WA caused the complete disappearance of the membrane. *T. mentagrophytes* 10340 colonized and significantly reduced the thickness of the bovine membranes only in WA. The thickness decrease shown by *T. rubrum* (1000A and 10318) was not statistically significant (two-sample paired *t*-test; *p* > 0.05), probably due to the lack of complete membrane coverage by these two strains.

Mechanical characterization of the membranes was evaluated by measuring the Firmness Index (FI) before and after the fungal colonization. In this way it can be noticed a substantial difference in compactness: FI was 72.79 ± 22.70 before fungal colonization, whereas after, FI was 27.85 ± 14.48, showing a reduction in compactness. The high standard deviation is due to the different morphology of the chosen membranes and the different thicknesses of the hoof. FI was normalized to the thickness of the membranes. FI values were measured on the membranes placed in ½ SDA, except for *T. rubrum* (1000A), and only the area where the fungus colonized the membrane was considered. WA was not evaluated as most of the membranes were highly degraded by three out of five strains tested (Figure 9b).

*T. rubrum* showed the highest degradation in the colonized area, as the FI values were 7.20 (10318) and 14.05 (1000A).

On the other hand, *T. interdigitale* (10379) and *T. mentagrophytes* (10326 and 10340) showed similar behavior with FI values of 32.53, 31.66, and 40.69, respectively.

#### 3.4.3. SEM

SEM photographs allowed us to observe tested fungal strains’ growth capacity on the bovine membrane. The three-dimensional images showed the membrane colonization kinetic, allowing observation of how pathogens colonized and exploited the membrane surface and how the hyphae were able to penetrate the membrane structure.

**Figure 10:**
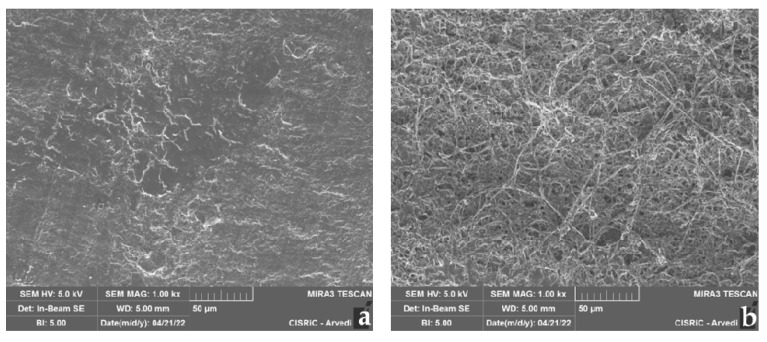
SEM image of control membrane without fungal growth (**a**); SEM image of *T. mentagrophytes* 10326 mycelium on bovine membrane after 27 days (**b**).

**Figure 11:**
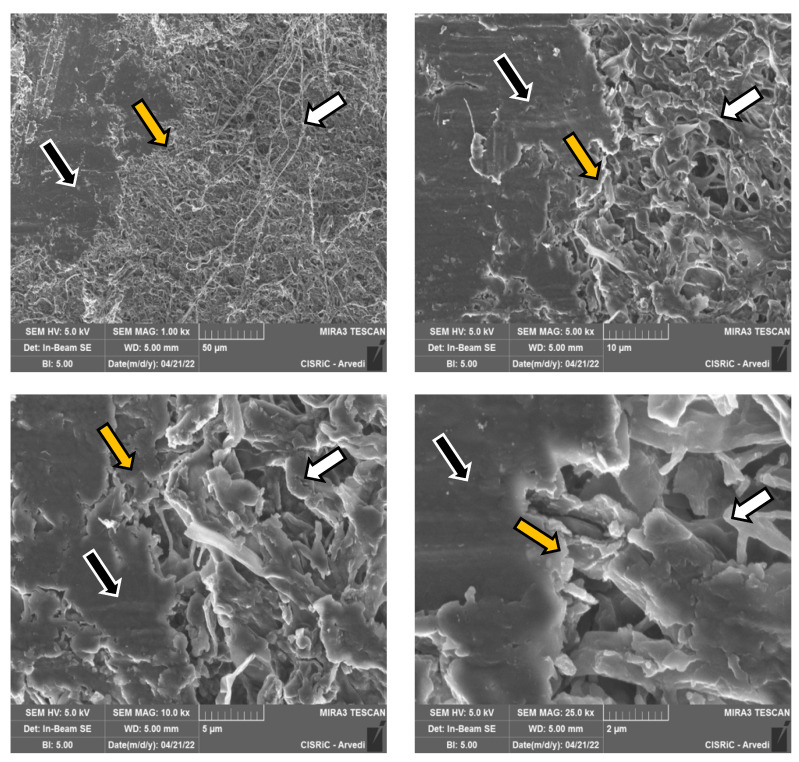
SEM images of colonization and growth on membrane by *T. mentagrophytes* 10326, in different magnifications. In each image, a membrane portion not yet colonized is visible on the **left** (black arrows), and the already developed mycelium on the **right** (white arrows). In the center, there is the point of contact between the fungus and the membrane, which at higher magnifications can be seen to be degraded (yellow arrows).

As an example, only the case of *T. mentagrophytes* 10326 was reported. SEM images captured the bovine membrane without infection (control) in its normal aspect (Figure 10a) and the membrane colonized by the strain, with the production of dense mycelium characterized by branched hyphae (Figure 10b). In Figure 11, the contact point between mycelium and membrane at different magnifications was observed.

## 4. Discussion

In the present study, 96 nail samples were examined, and 40 etiological agents were isolated. Of these, 60% belonged to yeasts, 22.5% to dermatophytes, and 17.5% to other filamentous fungi. Dermatophytes were only isolated from toenails. Papini et al. [9] stated that onychomycosis epidemiology in Italy is still unclear; they have diagnosed dermatophytes in almost 77% of the cases, 17% in yeast, and 6% in other filamentous fungi. Studies in different geographical areas have also reported more dermatophyte infections than non-dermatophyte filamentous fungi and yeasts in toenails [38,39,40].

It must be considered that for the present work, nails were collected and analyzed during the Coronavirus Disease 2019 (COVID-19) pandemic period, which involved many restrictions for the population. The inability to frequent places with a high risk of infection, such as sports centers, swimming pools, and beauty and wellness centers [41,42], may have decreased the number of dermatophyte infections.

Among the dermatophytes isolated, the most abundant species was *T. rubrum*, which accounted for 66% of infections. This percentage is consistent with the literature, according to which *T. rubrum* is the cause of most onychomycosis by dermatophytes [42,43,44,45,46], with a higher infection rate (>50%) than *T. mentagrophytes/T. interdigitale* group (about 20%) [47,48,49,50]. The nine *Trichophyton* strains isolated were used to evaluate the dermatophytes capacity to colonize bovine membranes, used as in vitro nail models for onychomycosis. Initial growth tests were performed to evaluate the best cultural media for the subsequent tests.

In particular, three cultural media with different nutrient concentrations were set up: WA, SDA, and ½ SDA. Results of growth tests showed the complete coverage of Petri dishes in rich media regardless of the concentration of SDA (complete or half). The low difference in strain growth between SDA and ½ SDA led to the exclusion of the culture medium with the highest nutrient concentration (SDA). All the strains grew in minimal medium (WA) too.

Hence, WA and ½ SDA media were selected to better understand the *Trichophyton* colonization kinetics on bovine membranes. WA was used both to force dermatophyte strains to colonize bovine membranes (being keratin the main source of nutrients) and at the same time to reduce the influence of the culture medium [17,51,52]. Similar observations were also reported by Valkov et al. [53] and Reichl and Müller-Goymann [54]. The authors used rich media, respectively the Yeast Medium (glucose, malt extract, peptone, and yeast extract) and Potato Glucose Agar, and noticed dermathophytes tend to utilize peptone dissolved in the rich medium, which is more easily metabolized than the ungual keratin. Moreover, Kunert [20] reported that dermatophytes are adapted to use proteins, peptides, and amino acids as the main nutritional source, even in the presence of simple sugars.

To value the membrane colonization by the nine *Trichophyton* strains, first, the ability of the strains to make contact with bovine membranes was observed, and subsequently their growth.

It was observed that two *T. rubrum* strains (10356 and 10380) did not get in touch with bovine membranes and two others (1000B and 10351) were not able to create initial visible contact with the membrane in minimal cultural media. Therefore, they were excluded from subsequent analyses aimed at evaluating changes in the bovine membrane after fungal colonization. *T. interdigitale* (10379) and *T. mentagrophytes* (10326) covered 100% of the bovine membranes placed in WA, whereas for *T. mentagrophytes* (10340), membrane coverage was the same with the two cultural media (WA and ½ SDA). *T. rubrum* covered only 25% of bovine membranes: strain 1000A in WA strain 10318 in ½ SDA.

Even if the incidence of cases of onychomycosis due to *T. rubrum* was reported to be higher than the *T. interdigitale/T. mentagrophytes* complex [55,56], the latter was more aggressive than *T. rubrum* [57]; this was probably due to the differences in enzyme expression of the two dermatophyte species [58]. Indeed, it is reported that *T. mentagrophytes* produces more keratinolytic enzymes than *T. rubrum* [50,59].

Mechanical characterization was observed by evaluating the wettability through the measurement of the contact angle (CA), the reduction of the bovine membrane thickness, and the firmness index (FI), which expresses the cohesion degree of the corneocytes that constitute the nail plate. Each test was performed before and after fungal colonization.

All the bovine membranes tested (with or without fungi) were not very wettable, being CA 71.08 ± 12.07 before and 81.06 ± 16.95 after fungal colonization. Additionally, the membranes had the same trend over time. Obtained results are consistent with those reported in similar cases [29]. The composition and structure of the bovine membranes justify the poor wettability [60].

As concerns membrane thickness, *T. interdigitale* and *T. mentagrophytes* significantly reduced colonized membranes thickness both in WA and ½ SDA (except for *T. mentagrophytes* 10340 only in WA). The strain *T. mentagrophytes* 10326 has completely degraded the membrane after 27 days in WA. *T. rubrum* showed no statistically significant difference in membrane thickness decrease. These data can be correlated with the scarce colonization (25%) of bovine membranes by the two tested strains (1000A and 10318). Membrane thickness evaluation is considered essential as it can influence permeability to antifungal drugs for onychomycosis treatment [60]. As evidenced by the data obtained with Firmness Index (FI), also the compactness of the membranes resulted in modified before and after the fungal colonization: FI passed from 72.79 ± 22.70 to 27.85 ± 14.48. It must be noted that it was impossible to analyze the whole membrane as some sections were completely degraded or were inaccessible due to the fungal presence, that in a few cases, was able to grow and penetrate within the membrane itself, as visible from the SEM images (Figure 10).

This can also explain the high standard deviation. Considering each fungal species, *T. rubrum* showed the highest degradation limited to the small area colonized (7.20 for 10318 and 14.05 for 1000A) but also *T. interdigitale* and *T. mentagrophytes* showed a reduction in compactness of about 50%.

One of the main novelties of this work was precisely the use of mechanical characterization of membranes thanks to the NailStress Meter NM 100 instrument, patented by the laboratory itself [36]. In this way, it was possible to calibrate the thickness of the membranes and their structural characteristics, as each type of experiment needs different families of membranes.

## 5. Conclusions

In conclusion, the present study revealed different colonization capacities for nine *Trichophyton* strains tested on bovine membranes, used as an in vitro model. Membranes were placed in Petri dishes containing culture medium, and, in general, the minimal one (water agar) proved to be the best for observing fungal colonization of the membranes themselves. The intention was to force the dermatophyte strains to colonize keratin as a source of nutrients and not the cultural medium. This should mimic what happens during an onychomycosis attack by the dermatophytes.

The procedure used yielded very interesting results in assessing the ability of *Trichophyton* to colonize and degrade membrane keratin, allowing us to evaluate not only the aggressiveness of different species but also the differences among strains belonging to the same species.

Results obtained from this work represent a starting point for the definition of a therapeutic and personalized diagnostic study to use this protocol in clinical investigations. This in vitro system could also be very useful in the future for assessing the efficacy of antifungal treatments, resulting in more appropriate and effective care for the patients.

## Figures and Tables

**Table 1:** Analysis parameter of Nail StrainStress Meter-NM100.

Start Position	2.3 mm
Step Size	0.001 mm
Start Force	0.10 N
Stop Force	6 N
Deflection	10 mm

**Table 2:** *Trichophyton* species identified with corresponding codes. (Medical mycology strain collection, MicUNIPV, DSTA, University of Pavia).

Code	Species
1000A	*T. rubrum*
1000B	*T. rubrum*
10318	*T. rubrum*
10351	*T. rubrum*
10356	*T. rubrum*
10380	*T. rubrum*
10379	*T. interdigitale*
10326	*T. mentagrophytes*
10340	*T. mentagrophytes*

## Data Availability

Not applicable.
